# The Complete Mitochondrial Genome of *Aix galericulata* and *Tadorna ferruginea*: Bearings on Their Phylogenetic Position in the Anseriformes

**DOI:** 10.1371/journal.pone.0109701

**Published:** 2014-11-06

**Authors:** Gang Liu, Lizhi Zhou, Bo Li, Lili Zhang

**Affiliations:** 1 Institute of Biodiversity and Wetland Ecology, School of Resources and Environmental Engineering, Anhui University, Hefei, Anhui, P. R. China; 2 Anhui Biodiversity Information Center, Hefei, Anhui, P. R. China; RIKEN Advanced Science Institute, Japan

## Abstract

*Aix galericulata* and *Tadorna ferruginea* are two Anatidae species representing different taxonomic groups of Anseriformes. We used a PCR-based method to determine the complete mtDNAs of both species, and estimated phylogenetic trees based on the complete mtDNA alignment of these and 14 other Anseriforme species, to clarify Anseriform phylogenetics. Phylogenetic trees were also estimated using a multiple sequence alignment of three mitochondrial genes (Cyt b, ND2, and COI) from 68 typical species in GenBank, to further clarify the phylogenetic relationships of several groups among the Anseriformes. The new mtDNAs are circular molecules, 16,651 bp (*Aix galericulata*) and 16,639 bp (*Tadorna ferruginea*) in length, containing the 37 typical genes, with an identical gene order and arrangement as those of other Anseriformes. Comparing the protein-coding genes among the mtDNAs of 16 Anseriforme species, ATG is generally the start codon, TAA is the most frequent stop codon, one of three, TAA, TAG, and T-, commonly observed. All tRNAs could be folded into canonical cloverleaf secondary structures except for tRNASer (AGY) and tRNALeu (CUN), which are missing the "DHU" arm.Phylogenetic relationships demonstrate that *Aix galericula* and *Tadorna ferruginea* are in the same group, the Tadorninae lineage, based on our analyses of complete mtDNAs and combined gene data. Molecular phylogenetic analysis suggests the 68 species of Anseriform birds be divided into three families: Anhimidae, Anatidae, and Anseranatidae. The results suggest Anatidae birds be divided into five subfamilies: Anatinae, Tadorninae, Anserinae, Oxyurinae, and Dendrocygninae. Oxyurinae and Dendrocygninae should not belong to Anserinae, but rather represent independent subfamilies. The Anatinae includes species from the tribes Mergini, Somaterini, Anatini, and Aythyini. The Anserinae includes species from the tribes Anserini and Cygnini.

## Introduction

Anseriformes is a highly differentiated order of birds with worldwide distribution, containing more than 150 species [1], [2]. Anseriformes is one of the best-studied groups of birds, largely owing to the group's historic importance in hunting, domestication, and aviculture [3]. The phylogenetic relationships among the Anseriformes, especially the phylogenetic position of several important species and groups, are rather complex and controversial, and have been affected by rearrangements several times throughout history [1], [2], [3].

Mandarin duck (*Aix galericulata*) and Ruddy shelduck (*Tadorna ferruginea*) are two typical Anseriform waterfowl, yet their phylogenetic status has remained controversial [3]. Traditionally, *Aix galericulata* belongs in Tadornini, and *Tadorna ferruginea* is a member of Cairinini; both of them placed inside Anatinae. The two birds have a moderately large body size, contrastingly pale dorsal wing-coverts, and blunt carpal (wing) spurs, along with other shared morphological characteristics; and both also feed through a combination of wading and dabbling [3]. All of these characteristics lie between the true ducks (Anatinae) and true geese (Anserinae) in terms of anatomy and behavior [12]. Accordingly, some authors believe *Aix* and *Tadorna* form an independent lineage together, the subfamily Tadorninae [8]. However, this view has often been challenged, and relationships within Tadorninae/Tadornini remain problematic, in particular, the taxonomy and systematic relationships within the groups. Many authors support *Cairina* a so-called “perching duck”, and *Aix* clustering together as a sister group, belonging to Tadornini [1], [2], [3]. Others suggest that *Aix* and *Cairina* should to be placed in their own tribe, Cairinini, within Anatinae [6]. However, still other authors consider the genera *Aix* and *Cairina* as forming the subtribe Cairinina, placed in the tribe Anatini [9]. Even other authors suggest that Tadornini (containing *Aix* and *Cairina*) should not belong to Anatinae at all, but rather represents the independent subfamily Tadorninae, placed in Anatidae [3]. Researchers have also used molecular evidence, based on combined Cyt b and ND2 gene sequences, to suggest placing *Aix* and *Tadorna* together as a tribe in the Anatinae [2]. These authors have also grouped the shelducks (*Tadorna*) together with the sheldgeese (*Cyanochen*, *Alopochen*, *Neochen* and *Chloephaga*), forming the tribe Tadornini, based on morphological and molecular data, but consider it a non-monophyletic grouping [2]. To add to the confusion, traditional primitive characters used to define “perching ducks” describes a polyphyletic grouping, because similar morphological, biochemical, and behavioral characteristics occur in many differing genera, including *Anseranas*, *Dendrocygna*, *Sarkidiornis*, *Tadorna*, *Cairina*, *Aix*, and *Chenonetta* [13].

Nonetheless, important groups in the Anseriformes remain controversial. Traditionally, the order Anseriformes has been considered to be composed of the families Anhimidae (two genera and three species) and Anatidae (approximately 41 genera and 147 species, including *Anseranas semipalmata*) [1]. However, some authors suggest that it should be divided into three families: Anhimidae, Anatidae, and Anseranatidae, the latter only containing one species, *Anseranas semipalmata* [1], [2]. The family Anhimidae is supported by both morphological and molecular data, usually without controversy [1], [3]. A major source of conflict at the family level is centered around *Anseranas semipalmata*, that is, whether it should be considered a member of an independent family by itself, or whether it is contained within a subfamily of Anatidae [1], [2], [4], [5], [6]. The Anatidae comprises the largest number of species in Anseriformes, and traditionally was divided into two subfamilies: Anatinae and Anserinae [1], [2], [3], [7], [8]. However, this view has been challenged by several authors, who recognize five subfamilies within Anatidae: Anatinae, Anserinae, Oxyurinae, Dendrocygninae, and Anseranatinae [2], [9], [10]. Anatinae, Anserinae, and Dendrocygninae are supported by previously published mtDNA data [10]. The stiff-tailed ducks (Oxyurinae/Oxyurini) include some of the most distinctive waterfowl species, showing the greatest sexual size dimorphism [11]. Most of its members have long stiff-tail feathers, which are erected at rest, and relatively large, swollen bills [3]. According to morphological and behavioral characteristics, stiff-tailed ducks (Oxyurinae/Oxyurini) appear to be closer to swans and true geese than they are to typical ducks [3]. Previously the group was considered to be a comparatively primitive member of the tribe Oxyurini; however, some authors do not support this view and considered it to be a subfamily, Oxyurinae [3]. Its relationships are still enigmatic, and are subject to considerable debate regarding its validity and circumscription [3], [11]. Systematic controversies concerning the stiff-tailed ducks (Oxyurinae/Oxyurini) have focused on whether they constitute a tribe or a subfamily, and often consider it to be close to Anserinae, not within Anatinae at all, and agree with morphology-based studies [8], [9], [11].

Mitochondrial DNA is a powerful, increasingly popular, and widely used molecular marker for the estimation of the animal phylogenetic relationships. It has become a major tool of comparative genomics and plays an important role in phylogenetic studies, comparative and evolutionary genomics, and molecular evolutionary analyses, owing to its maternal inheritance, lack of recombination, and accelerated nucleotide substitution rates compared with those of the nuclear DNA [14], [15]. Here, we attempt to resolve the controversial Aves species using mtDNA analyses. Our newly completed mitochondrial genomes should provide new insights into the phylogenetic position of important species, and yield insight into the higher-level systematics of Anseriform birds. Early molecular work disentangling the phylogeny of the Anseriformes was mostly based on one or a few mitochondrial loci, almost always including Cyt b, ND2, and/or control region (CR) sequences [1], [2]. However, complete mtDNA sequences have become increasingly important for comprehensive evolutionary and phylogenetic studies [10], [15], [16]. Several analyses have demonstrated that complete mtDNA provides higher levels of phylogenetic support than those based on individual or partial mitogenomes [10], [15], [16], [17], [18], [19]. Complete mtDNA sequences are not only more informative than shorter sequences of individual genes, but also provide reliable information toward the inference of phylogenetic relationships among controversial animals [17], [19], [20], [21]. Consequently, complete mtDNA genomes are becoming a preferred marker for resolving controversial species relationships, and are increasingly important for comprehensive evolutionary studies [10], [14], [15], [16], [22]. However, very few Anseriform birds are currently represented with complete mtDNAs, consequently, a number of Anseriform species and their phylogenetic relationships remain unresolved. We sequenced the complete mtDNA of two important Anseriform birds, from the genera *Aix* and *Tadorna*, in this study. We also analyzed the nucleotide composition, codon usage, and compositional biases of the mitogenomes. Our phylogenomic analysis should shed increased light on the phylogenetic status of *Aix galericulata* and *Tadorna ferruginea*, and on the phylogenetic relationships of other important groups of Anseriformes.

## Results

### Genome organization and arrangement

The complete mtDNAs of *Aix galericulata* and *Tadorna ferruginea* are 16,651 and 16,639 bp in length, respectively. Both contain the typical set of 37 genes, including 13 protein-coding genes (PCGs) (ATP6, ATP8, COI-III, ND1-6, ND4L, and Cyt b), two rRNAs (12S rRNA and 16S rRNA), 22 tRNAs and a putative CR (D-loop) (Table 1). The heavy (H-) DNA strand carries most of the genes, 12 PCGs, two rRNAs, and 14 tRNAs. ND6 and eight tRNAs are located on the light (L-) strand (Table 1).

**Table 1:** Organization of the complete mtDNAs of *Aix galericulata* and *Tadorna ferruginea*.

Gene	Direction	*Aix galericulata*				*Tadorna ferruginea*			
		Nucleotide no	Size	Start codon	Stop codon	Nucleotide no	Size	Start codon	Stop codon
D-loop	F	1–1071	1071			1–1077	1077		
tRNA^Phe^	F	1072–1139	68			1078–1147	70		
12S rRNA	F	1140–2136	997			1148–2129	982		
tRNA^Val^	F	2137–2208	72			2130–2200	71		
16S rRNA	F	2209–3812	1604			2201–3810	1610		
tRNA^Leu^	F	3813–3886	74			3811–3884	74		
ND1	F	3893–4870	978	ATG	AGG	3891–4868	978	ATG	AGG
tRNA^Ile^	R	4869–4941	73			4867–4939	73		
tRNA^Gln^	F	4949–5019	71			4947–5017	71		
tRNA^Met^	F	5019–5087	69			5017–5085	69		
ND2	F	5088–6128	1041	ATG	TAA	5086–6126	1041	ATG	TAG
tRNA^Trp^	R	6127–6203	77			6125–6199	75		
tRNA^Ala^	R	6207–6275	69			6203–6271	69		
tRNA^Asn^	R	6276–6353	78			6274–6346	73		
tRNA^Cys^	R	6354–6418	65			6347–6411	65		
tRNA^Tyr^	F	6419–6492	74			6412–6482	71		
COI	R	6494–8044	1551	GTG	AGG	6484–8034	1551	GTG	AGG
tRNA^Ser (UCN)^	F	8036–8108	73			8026–8098	73		
tRNA^Asp^	F	8111–8179	69			8101–8169	69		
COII	F	8181–8867	687	GTG	TAA	8171–8857	687	ATG	TAA
tRNA^Lys^	F	8869–8937	69			8859–8927	69		
ATP8	F	8939–9106	168	ATG	TAA	8929–9096	168	ATG	TAA
ATP6	F	9097–9780	684	ATG	TAA	9087–9770	684	ATG	TAA
COIII	F	9780–10563	784	ATG	T-	9770–10541	772	ATG	T-
tRNA^Gly^	F	10564–10632	69			10542–10610	69		
ND3	F	10633–10984	352	ATG	TAG	10611–10962	352	ATG	TAG
tRNA^Arg^	F	10986–11055	70			10964–11033	70		
ND4L	F	11056–11352	297	ATG	TAA	11034–11330	297	ATG	TAA
ND4	F	11355–12723	1369	ATT	T-	11333–12701	1369	ATT	T-
tRNA^His^	F	12724–12792	69			12702–12770	69		
tRNA^Ser^ ^(AGY)^	F	12793–12859	67			12771–12836	66		
tRNA^Leu (CUN)^	F	12859–12929	71			12836–12907	72		
ND5	F	12930–14756	1827	GTG	TAA	12908–14728	1821	GTG	TAA
Cyt b	F	14756–15898	1143	ATG	TAA	14728–15870	1143	ATG	TAA
tRNA^Thr^	R	15901–15969	69			15872–15940	69		
tRNA^Pro^	R	15982–16051	70			15951–16020	70		
ND6	R	16062–16583	522	ATG	TAG	16031–16552	522	ATG	TAG
tRNA^Glu^	F	16584–16651	68			16553–16639	87		

### Protein-coding genes

Through the 13 protein-coding genes, ATG is the start codon in nine of the 13 PCGs in *Aix galericulata*, but ND4 starts with ATT, while COI, COII, and ND5 begin with the nonstandard start codon GTG. In *Tadorna ferruginea* ten PCGs start with ATG, and ATT is the start codon only in ND4, while COI and ND5 begin with GTG. The standard stop termination codon TAA occurs in most of the same genes in the two birds' mtDNAs, except ND2 stops with TAA in *Aix galericulata* and TAG in *Tadorna ferruginea*. Furthermore, AGG terminates the ND1 and COI genes, TAG terminates the ND3 and ND6 gene, and the incomplete termination codon T- occurs in the COIII and ND4 genes in both birds.

### Ribosomal RNA, transfer RNA, and non-coding regions

In *Aix galericulata* and *Tadorna ferruginea* 12S rRNA (997 bp and 982 bp, respectively) and 16S rRNA (1,604 bp and 1,610 bp, respectively) genes are located between the tRNA^Phe^ and tRNA^Leu^ genes, separated by the tRNA^Val^ gene. The two complete mtDNAs contain 22 tRNAs genes, and except for tRNA^Ser (AGY)^ and tRNA^Leu (CUN)^, which lack dihydrouridine (DHU) arms, all other tRNAs could be folded into the typical cloverleaf structure. The longest tRNAs are tRNA^Asn^ (78 bp) and tRNA^Glu^ (87 bp) in *Aix galericulata* and *Tadorna ferruginea*, respectively, and the shortest is tRNA^Cys^ (65 bp in both).

Non-coding regions in the mtDNAs include the CRs and a few intergenic spacers. The CRs are located between the tRNA^Glu^ and tRNA^Phe^ genes, which are 1,071 bp and 1,077 bp, respectively, in *Aix galericulata* and *Tadorna ferruginea*. Additionally, 11 gene junction regions spacer by a total of 31 bp, with the longest one being 10 bp between ND6 and tRNA^Glu^ in *Aix galericulata* (Table 1). There are a total of 55 bp spacer region at 12 gene junctions in *Tadorna ferruginea* (Table 1).

### Phylogenetic reconstructions

**Figure 1:**
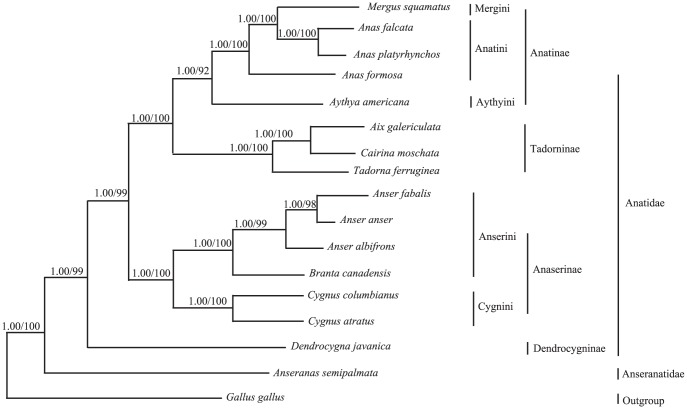
Phylogenetic relationships among the 16 Anseriforme species based on complete mtDNA sequences. Numbers at each node are Bayesian posterior probabilities (left) and maximum likelihood bootstrap proportions (estimated from 100 pseudoreplicates) (right).

Our chosen 16 Anseriforme species represent two major branches of the Anseriformes phylogeny with highly similar topologies and only slight differences in bootstrap support and posterior probability values (Figure 1). The first branch is Anatidae and the second is Anseranatidae. Anatidae contains Anatinae, Tadorninae, Anserinae, and Dendrocygninae; and Anseranatidae only contains *Anseranas semipalmata*. Anatinae and Tadorninae are sister groups, grouped together nestled within the clades Anserinae and Dendrocygninae. Mergini, Anatini, and Aythyini form Anatinae; Anserinae contains Anserini and Aythyini. *Aix galericulata* and *Tadorna ferruginea* are in the Tadorninae group.

**Figure 2:**
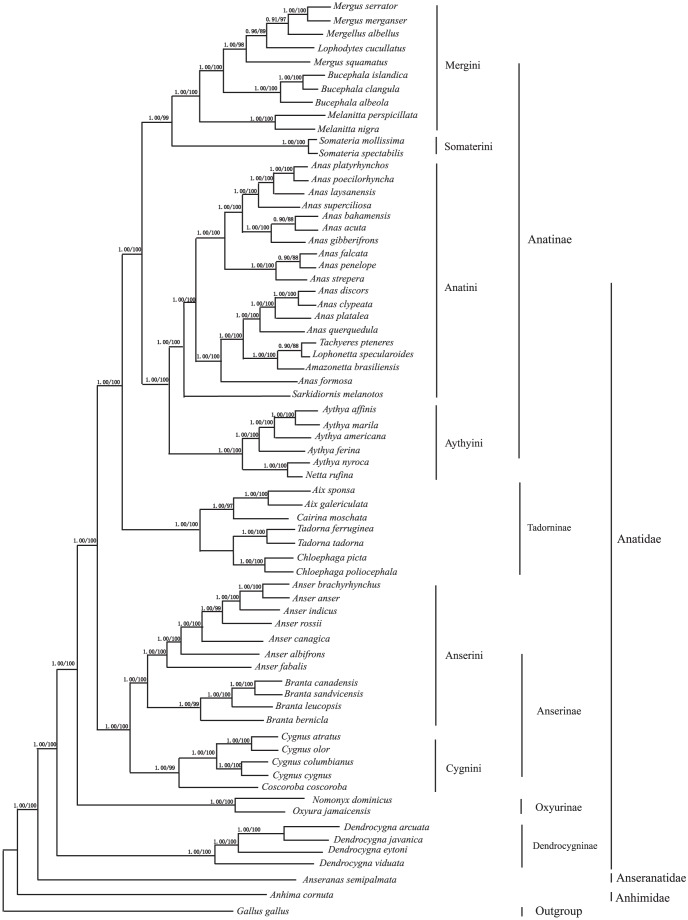
Phylogenetic relationships among the 68 Anseriforme species based on Cyt b, ND2, and COI genes. Numbers at each node indicate Bayesian posterior probabilities (left) and maximum likelihood bootstrap proportions (from 100 pseudoreplicates) (right).

Phylogenetic analysis was also performed on a concatenated Cyt b, ND2, and COI genes among 68 Anseriforme species. The trees from the maximum likelihood (ML) and Bayesian inference (BI) analyses share identical topologies and high node support values (Figure 2). The results indicate that Anseriformes could be divided into three branches: Anatidae, Anseranatidae, and Anhimidae. Anatidae and Anseranatidae are sister branchs, then grouped with Anhimidae. Anatidae contains five clades: Anatinae, Tadorninae, Anserinae, Oxyurinae, and Dendrocygninae. The Anatinae, includes species from the tribes Mergini, Somaterini, Anatini, and Aythyini. This subfamily is a sister group to Tadorninae, comprising *Aix*, *Cairina*, *Tadorna*, and *Chloephaga*. These two subfamilies form a clade that is in a sister group relationship with Anserinae, comprising the tribes Anserini and Cygnini. In turn, this clade is sister to the remaining Oxyurinae and Dendrocygninae. All of them are grouped with families Anseranatidae and Anhimidae.

## Discussion

### Mitochondrial genome annotation and features

The gene order and arrangement of the two new mtDNA sequences, including such features as gene length, base composition, and RNA structure, are extremely conservative, and similar to that of other Anseriform birds [10], [16], [23], [24]. The overall base composition is similar to other Anseriforme species, for example, A+T content is higher than C+G content, conforming to other Anseriforme species (51.6–55.7%) [10], [16], [23], [24]. The relative abundance of nucleotides is C>A>T>G, reflecting the strong AT bias [10], [16]. Guanine is the rarest nucleotide.

Results indicate that all Anseriformes mtDNAs so far sequenced have the same gene order and arrangement, no introns, no long intergenic spacers, and only a few overlapping sequences [10], [16]. All genes are encoded on the same arrangement, and there are no missing or duplicated genes [10], [16]. Among the 16 mtDNAs the longest is from *Anseranas semipalmata* (16,870 bp), and the shortest is *Anas formosa* (16,594 bp). Homologous regions comprise 12,748 bp, representing 79.68% of the complete genome (Table 2). The 16 genomes generally have the highest transition/transversion ratio in closely-related species [25]. The A, T, and A+T compositions are similar, and shared with a strong AT bias and rare guanines (Table 2). Metazoan mtDNA usually present a clear strand bias in nucleotide composition; this strand bias can be measured as AT-skew and GC-skew [26]. All of the Anseriformes mtDNAs exhibit a slight AT-skew (average value: 0.137), ranging from 0.125 in *Dendrocygna javanica* to 0.147 in *Tadorna ferruginea* (Table 2). The GC-skew ranges from −0.377 (*Anseranas semipalmata*) to −0.327 (*Dendrocygna javanica*), with an average value of −0.353 (Table 2).

**Table 2:** Nucleotide composition (%) of some Anseriforme mitochondrial genomes.

Species	T (%)	C (%)	A (%)	G (%)	A+T (%)	G+C (%)	AT-skew	GC-skew	Total nucleotide
*Anser albifrons*	22.63	32.05	30.15	15.18	52.78	47.23	0.142	−0.357	16 737
*Anser anser*	22.58	32.14	30.19	15.09	52.77	47.23	0.144	−0.361	16 738
*Anser fabalis*	22.74	31.84	30.06	15.36	52.80	47.20	0.138	−0.349	16 688
*Anas formosa*	22.51	32.44	29.52	15.53	52.03	47.97	0.134	−0.353	16 594
*Anas platyrhynchos*	22.19	32.52	29.21	16.08	51.40	48.60	0.136	−0.338	16 606
*Aythya americana*	22.24	32.75	29.39	15.62	51.60	48.40	0.138	−0.354	16 616
*Anseranas semipalmata*	23.49	31.38	30.92	14.21	54.41	45.59	0.136	−0.377	16 870
*Branta canadensis*	22.60	32.07	30.18	15.15	52.78	47.22	0.143	−0.358	16 760
*Cygnus columbianus*	22.79	31.89	30.10	15.22	52.89	47.11	0.138	−0.354	16 728
*Cygnus atratus*	22.20	32.55	29.52	15.73	51.72	48.28	0.142	−0.348	16 748
*Cairina moschata*	21.93	32.95	29.00	16.12	54.88	45.12	0.129	−0.373	16 610
*Dendrocygna javanica*	23.67	30.44	30.44	15.45	54.11	45.89	0.125	−0.327	16 753
*Mergus squamatus*	22.26	32.76	29.04	15.94	51.30	48.70	0.132	−0.345	16 595
*Anas falcata*	22.35	32.65	28.89	16.11	51.24	48.76	0.128	−0.339	16 601
*Aix galericulatata*	22.36	32.65	29.24	15.75	51.60	48.40	0.133	−0.349	16 651
*Tadorna ferruginea*	22.00	32.96	29.57	15.47	51.57	48.43	0.147	−0.361	16 639

### Comparison of protein-coding genes

We compared the total length of the 13 PCGs in *Aix galericulata* and *Tadorna ferruginea* with other Anseriform birds. Lengths among them are quite similar and very conservative; the longest one is *Aix galericulata* (11,403 bp) and the shortest is *Anser fabalis* (11,328 bp). The 13 PCGs have a total length of 11,331 bp in *Aix galericulata* and 11,385 bp in *Tadorna ferruginea*, which is 68.05% and 68.42% of each entire mtDNA genome, respectively. In both species, the longest gene is ND5, located between the tRNA^Leu (CUN)^ and Cyt b genes, and the shortest is ATP8, which is between the tRNA^Lys^ and ATP6 genes. Most PCGs used ATG as start codons, only a few start with GTG, GTC, or ATA. Stop codons are also similar across species, with TAA, TAG, and T- occurring most frequently. In *Tadorna ferruginea* and *Tadorna ferruginea*, the start condons are ATG, GTG and ATT, and TAA, AGG, TAG and T- as stop termination codons occur in most of the same genes in the two birds' mtDNAs. Among the 13 PCGs, specific examples include the following: the COI initiation codon is GTG and the termination codon is AGG in all 16 species; Cyt b starts with ATG and ends with TAA; COII starts with ATG and ends with TAG, except in *Branta canadensis*, where it starts with GTC; ND6 starts with ATG and ends with TAG, except in *Anser fabalis* it ends with TAA; ND1 starts with ATG and ends with AGG, except in *Anser albifrons* where the stop codon is TAA; and ND2 starts with ATG and ends with TAG, except in *Anseranas semipalmata*, *Cygnus atratus*, and *Tadorna ferruginea*, where the stop codon is TAA (Table 3).

**Table 3:** Predicted initiation and termination codons for 13 mitochondrial PCGs in the 16 Anseriforme species.

Gene	Predicted initiation and termination															
	A	B	C	D	E	F	G	H	I	J	K	L	M	N	O	P
ND1	ATG/TAA	ATG/AGG	ATG/AGG	ATG/AGG	ATG/AGG	ATG/AGG	ATA/AGG	ATG/AGG	ATG/AGG	ATG/AGG	ATG/AGG	ATG/AGG	ATG/AGG	ATG/AGG	ATG/AGG	ATG/AGG
ND2	ATG/TAG	ATG/TAG	ATG/TAG	ATG/TAA	ATG/TAG	ATG/TAG	ATG/TAG	ATG/TAG	ATG/TAG	ATG/TAA	ATG/TAA	ATG/TAA	ATG/TAA	ATG/TAA	ATG/TAA	ATG/TAG
COI	GTG/AGG	GTC/AGG	GTG/AGG	GTG/AGG	GTG/AGG	GTG/AGG	GTG/AGG	GTG/AGG	GTG/AGG	GTG/AGG	GTG/AGG	GTG/AGG	GTG/AGG	GTG/AGG	GTG/AGG	GTG/AGG
COII	GTG/TAA	GTG/TAA	GTC/TAA	GTG/TAA	GTG/TAA	GTG/TAA	GTG/TAA	GTG/TAA	GTG/TAA	ATG/TAA	GTG/TAA	GTG/TAA	GTG/TAA	GTG/TAA	GTG/TAA	GTG/TAA
ATP8	ATA/TAA	ATG/TAA	ATG/TAA	ATG/TAA	ATG/TAA	ATG/TAA	ATG/TAA	ATG/TAA	ATT/TGG	AT G/TAA	ATG/TAA	ATG/TAA	ATG/TAA	ATG/TAA	ATG/TAA	ATG/TAA
ATP6	ATG/TAA	ATG/TAA	ATG/TAA	ATG/TAA	ATG/TAA	ATG/TAA	ATG/TAA	ATG/TAA	ATG/TAA	ATG/TAA	ATG/TAA	ATG/TAA	ATG/TAA	ATG/TAA	ATG/TAA	ATG/TAA
COIII	ATG/TAA	ATG/T-	ATG/TAA	ATG/T-	ATG/T-	ATG/T-	ATG/T-	ATG/T-	ATG/T-	ATG/T-	ATG/TAA	ATG/TAA	ATG/T-	ATG/TAA	ATG/T-	ATG/T-
ND3	ATG/TAG	ATG/TAA	ATG/TAA	ATG/TAG	ATG/TAA	ATG/AGG	ATG/TAA	ATG/TAA	ATG/TAA	ATA/TAA	ATG/T-	ATG/TAA	ATG/TAA	ATG/TAA	ATG/TAG	ATG/TAG
ND4L	ATG/TAA	ATG/TAA	ATG/TAA	ATG/TAA	ATG/TAA	ATG/TAA	ATG/TAA	ATG/TAA	ATG/T-	GTG/TAA	ATG/TAA	ATG/TAA	ATG/TAA	ATG/TAA	ATG/TAA	ATG/TAA
ND4	ATG/T-	ATC/T-	ATG/T-	ATG/T-	ATG/T-	ATG/T-	ATG/T-	ATG/TAA	ATG/T-	ATG/T-	ATG/TAA	ATG/TAA	ATG/TAA	ATG/TAG	ATG/T-	ATG/T-
ND5	GTG/TAA	GTG/AGA	AGT/TAA	GTG/AGA	GTG/AGA	GTG/TAA	GTG/TAA	GTG/TAA	GTG/TAA	ATG/AGA	GTG/AGA	GTG/TAA	GTG/AGA	GTG/TAA	GTG/TAA	GTG/TAA
Cytb	ATG/TAA	ATG/TAA	ATG/TAA	ATG/TAA	ATG/TAA	ATG/TAA	ATG/TAA	ATG/TAA	ATG/TAA	ATG/TAA	ATG/TAA	ATG/TAA	ATG/TAA	ATG/TAA	ATG/TAA	ATG/TAA
ND6	ATG/TAG	ATG/TAG	ATG/TAG	ATG/TAG	ATG/TAG	ATG/TAG	ATG/TAG	ATG/TAG	ATG/TAG	ATG/TAG	ATG/TAG	ATG/TAG	ATG/TAA	ATG/TAG	ATG/TAG	ATG/TAG

Notes: A: *Anser albifrons* (NC_004539), B: *Anser anser* (NC_011196), C: *Branta canadensis* (NC_0071011), D: *Cygnus atratus* (NC_012843), E: *Cygnus columbianus* (NC_007691), F: *Mergus merganser* (NC_016723), G: *Cairina moschata* (NC_010965), H: *Anas platyrhynchos* (NC_009684), I: *Aythya americana* (NC_000877), J: *Anseranas semipalmata* (NC_005933), K: *Dendrocygna javanica* (NC_012844), L: *Anas formosa* (NC_015482), M: *Anas fabalis* (NC_016922), N: *Anas falcata*, O: *Aix galericulata* (this study), P: *Tadorna ferruginea* (this study).

Some mtDNA PCGs are particularly worthy of note. Avian species generally exhibit moderate levels of sequence divergence in some mitochondrial genes, including Cyt b, ND2, and COI. These genes are of special interest, because they have been widely used to resolve the taxonomy of controversial groups in Anseriformes [2]. A combination of these three genes is often adequate and has been used for resolving phylogenetic problems at many different taxonomic levels, ranging from related species to genera and families [25]. They have been valuable for clarifying phylogenetic relationships within many controversial animal groups, especially that of Anseriform birds [16], [25], [27].

### Control region comparisons

The CR is the only major non-coding segment of mtDNA, and has higher variability, evolving three to five times more rapidly, than other vertebrate mtDNAs [28]. Its primary function is thought to be the regulation of replication and transcription [29]. In Aves the CR is located between the tRNA^Glu^ and tRNA^Phe^ genes. Sequence variation in the CR results in length variability in different birds [28], [29]. Similar to other Anseriform birds, there is only one CR in *Aix galericulata* and *Tadorna ferruginea*, 1,071 bp and 1,077 bp in length, respectively. Comparing the length of the CR in the 16 Anseriforme species that we researched shows lengths ranging from 1,045 bp (*Anas formosa*) to 1,335 bp (*Anseranas semipalmata*) with an average length of 1,127 bp. The high AT content (53.55%) is similar to other organisms [10], [16], [30]. Regions of recognizable homology comprise only 406 bp, representing 27.92% of the entire mtDNA genome. An alignment of the 16 CR sequences contains 406 variable sites and 537 parsimony informative sites. Its nucleotide frequencies are not significantly different from other birds. Some conspicuous macro-repeat units are found in the CR of some Anseriforme species, including microsatellite-like (C)_11_ repeats in *Anser albifrons* and *Anser anser*, and (ATCAAACG)_15_ elements in *Anseranas semipalmata*. The average genetic distance (0.376) between the CR of *Anseranas semipalmata* and the other species in our analysis is higher than average (0.288) (Table 4).

**Table 4:** The genetic distances among the 16 Anseriforme species of mtDNA CR based on kimura-2-parameter model in this study.

Genetic distances	1	2	3	4	5	6	7	8	9	10	11	12	13	14	15	16
1	—															
2	0.191	—														
3	0.203	0.065	—													
4	0.198	0.052	0.072	—												
5	0.329	0.262	0.264	0.274	—											
6	0.346	0.267	0.275	0.270	0.043	—										
7	0.331	0.269	0.274	0.281	0.027	0.062	—									
8	0.550	0.491	0.484	0.474	0.501	0.513	0.518	—								
9	0.219	0.142	0.146	0.135	0.266	0.264	0.278	0.493	—							
10	0.305	0.246	0.241	0.249	0.119	0.116	0.138	0.522	0.241	—						
11	0.171	0.153	0.143	0.150	0.295	0.309	0.301	0.496	0.153	0.275	—					
12	0.334	0.263	0.255	0.257	0.151	0.144	0.172	0.472	0.246	0.152	0.272	—				
13	0.318	0.260	0.254	0.259	0.148	0.141	0.167	0.513	0.245	0.147	0.258	0.102	—			
14	0.411	0.387	0.393	0.385	0.416	0.411	0.432	0.400	0.419	0.398	0.370	0.397	0.393	—		
15	0.184	0.187	0.182	0.183	0.328	0.340	0.330	0.533	0.223	0.292	0.178	0.327	0.309	0.407	—	
16	0.314	0.276	0.259	0.276	0.420	0.420	0.425	0.610	0.275	0.410	0.237	0.402	0.378	0.510	0.309	—

Notes: 1: *Aix galericulatata*, 2: *Anas falcata*, 3: *Anas formosa*, 4: *Anas platyrhynchos*, 5: *Anser albifrons*, 6: *Anser anser*, 7: *Anser fabalis*, 8: *Anseranas semipalmata*, 9: *Aythya americana*, 10: *Branta canadensis*, 11: *Cairina moschata*, 12: *Cygnus atratus*, 13: *Cygnus columbianus*, 14: *Dendrocygna javanica*, 15: *Mergus squamatus*, 16: *Tadorna ferruginea*.

**Figure 3:**
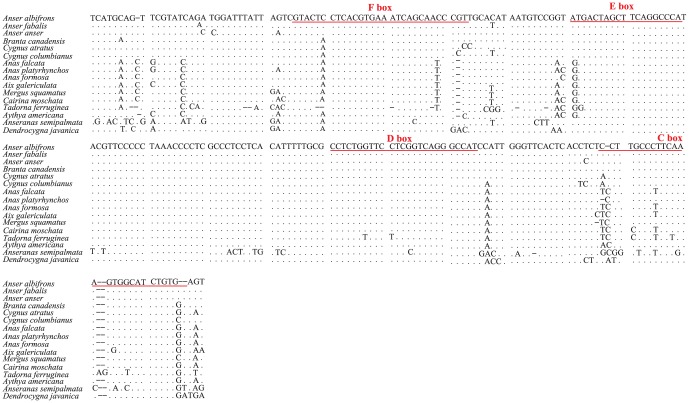
Structure of the central conserved domain of the CR in 16 Anseriforme species. Dots indicate nucleotides with identity to the reference sequences, and dashes indicate indels.

Comparative analysis of the structure and organization of CRs can help show relationships in the Anseriformes [8], [27], [31]. A better understanding of CR characteristics can provide insights into phylogenetics [28], *Anseranas semipalmata* thus has the most divergent CR of the studied species (Figure 3). Typically, vertebrate CRs are subdivided into three domains (Domain I, Domain II, and Domain III) [28]. Within the central conserved domain (Domain II), conserved sequence blocks (CSBs) C, D, E, and F show evolutionary conservation, and exhibit a rather homogeneous evolutionary rate [28], and are 31 bp, 24 bp, 20 bp and 20 bp in the length, respectively (Figure 3). It has lower variation rate among the Domain II of 16 Anseriforme species, and there are no base insert, only three bases deletion are found in *Aix galericulata* and 13 bases deletion in *Tadorna ferruginea* (Figure 3). These boxes are present in most avian taxa, are similar to those of other vertebrates, and are associated with regulating H-strand synthesis [8], [31]. Several CR CBS boxes exist in the 16 species of Anseriformes studied as well, which suggests that the boxes may play a key role in the replication and transcription of the mitochondrial genome [27], [31].

### Phylogenetic analyses

The trees from the ML and BI analyses based on the complete mtDNA of 16 Anseriform species share similar topologies and high node support values with those of the concatenated three mitochondrial gene sequences from 68 species (Figures 2 and 3). The results support the grouping of *Aix galericula* and *Tadorna ferruginea* together in the same lineage, Tadorninae. Livezey included *Aix* and *Cairina* in the Anatini but proposed a subtribe, Cairinina, clustering these species together on the basis of a single osteological synapomorphy [9]. Dickinson considered *Aix* and *Cairina* to be members of the Anatinae [6]. Our molecular results show *Aix* and *Cairina* grouped with *Tadorna* and *Chloephaga*, supported by high bootstrap values, forming the Tadorninae, which is located between Anatinae and Anserinae. Therefore, we agree that Tadorninae should be an independent subfamily in the Anatidae, and that *Aix* and *Cairina* don't belong to Anatinae, but are members of Tadorninae, which is a little different from Dickinson's view [6]. The taxonomy and systematic relationships within the Tadorninae have been considerably debated. Our results show that the relationships between *Aix* and *Cairina*, and *Tadorna* and *Chloephaga* are much closer. *Cairina* and *Aix* cluster together as a sister group adjacent to *Tadorna* and *Chloephaga*, which, based on morphological and molecular studies, has been repeatedly claimed [2]. *Aix* and *Cairina* have several similar characteristics in behavior and breeding biology, and molecular phylogenetic trees also suggest that they are sister branches, showing they have a close genetic relationship as well [8]. Our mtDNA evidence also supports *Tadorna* and *Chloephaga* having a much closer genetic relationship, congruent with morphological studies [9].

Our results combine the data of several new mitogenomes within the growing phylogeny of the Anseriformes. Molecular phylogenetic results indicate that the 68 Anseriform birds studied could be divided into three families: Anhimidae, Anatidae, and Anseranatidae. Anhimidae diverged first, followed by Anseranatidae and Anatidae, similar to previous molecular phylogenetic and morphological studies [1], [2], [4], [5]. *Anseranas semipalmata* does not belong in Anatidae, but represents an independent family, Anseranatidae [1], [2]. According to our study Aythyini diverged earlier than Anatini within Anatidae and is monophyletic [1], [2]. Systematic relationships concerning the stiff-tailed ducks (Oxyurinae) have also been very controversial [5], [8], [9]. According to traditional morphological studies the stiff-tailed ducks share a common ancestor with geese and swans [3], [9], are closest to the Anserinae, and are not within the Anatinae. Our results don't support stiff-tailed ducks (Oxyurinae) as being a tribe within Anatidae, but present them as an independent subfamily, Oxyurinae, which diverged earlier than the whistling ducks (Dendrocygninae) in Anatidae [2], [10], [16].

Our mtDNA analysis suggests that the genus *Anas* is actually polyphyletic, but *Anas formosa formosa*, which is found in one of the Anatini branches, has no close relatives among living ducks, and should be placed in some distinct genera. *Anas formosa formosa* should be in a distinct genus; and *Anas discors*, *Anas querquedula*, *Anas clypeata* and *Anas platalea* should be placed in another distinct genus, closest to the multigenus duck group (*Tachyeres*, *Lophonetta*, and *Amazonetta*).

## Materials and Methods

### Sample collection

Trace blood samples from *Aix galericulata* and *Tadorna ferruginea* were collected using non-invasive methods at the Hefei Wild Animal Park in May 2013. No animal was killed for the purpose of the experiment, the method will not affect the health of the animals, and it conforms to our animal ethics committee's guidline in this study. The Hefei Wild Animal Park is authorized to administer animal rescue and medical treatment by Anhui Provincial Conservation and Mangement Station for Wildlife (APCMSW), a provincial government agency for wildlife conservation in the Anhui Province of China. We were authorized to study the birds by the APCMSW. The samples were stored at −20°C at the Institute of Biodiversity and Wetland Ecology, School of Resources and Environmental Engineering, Anhui University (Sample codes are AHU-WB20130522 and AHU-WB20130523, respectively).

### DNA extraction, PCR amplification, and sequencing

Whole genomic DNA was isolated from blood samples using the phenol/chloroform method. Extracted DNA was examined on a 1.0% agarose/TBE gel and stored at −20°C as templates for PCR.

Based on an alignment of complete mtDNA sequences from *Cygnus atratus* (NC_012843), *Anas platyrhynchos* (NC_009684), and *Aythya americana* (NC_000877), we designed three primer pairs (primer sets 9, 11, and 14) using Primer 5.0. We also used other primers developed from *Anser fabalis* [10], [16]. These primers were used to amplify and sequence the complete mtDNAs of *Aix galericulata* and *Tadorna ferruginea* (Table 5). Generated sequences were all less than 1,200 bp each, with each segment overlapping the next by 80–150 bp.

**Table 5:** The 5 Primer sequences used in this study.

Primer No	Amplified region	Forward primer sequence (5'→3')	Reverse primer sequence (5'→3')
1	840–2106	CCACTACCCGAGACCTACG	TAAGTCTTTTGTCCGCAGGCAT
2	1997–3190	ATAGGGCTATTTAGTGAATGCT	TGAGTATTCTAAGTACACCTTC
3	3017–5121	GTCACCCTCCTCATAAGCCA	GTTGCTGGAGATTGTGATTGT
4	4944–7091	TTACCAAAAACATAGCCTTCAG	GTTGCTGGAGATTGTGATTGT
5	6886–8340	GATCAAAACTCTCCATACTTCC	TCGGTCGGTTAGTAGCATTGT
6	8210–9346	TGGCTATCTTCTCACTTCACCT	GGGTAGGATTGTTCAGATTAGT
7	9176–10285	ACCACGCTCTGATTGTTGCCT	TTGTGTGGTGGGGTGAATGT
8	10127–11367	CGACTTTCCACCATCCAACT	CTTCGTGGTATTCTATTGCCT
9	11017–12122	TACTAAACACAGCAATCCTCCT	GAATTTTGGTGGGGACAGTAG
10	11944–12925	CTACACCTGAGCTTCTACT	GAGACAGATTGAGCTAGT
11	12747–13834	TCTGACTACCAAAAGCCCAC	GTGAGTAGTGTGAGGGAGTT
12	13685–14810	GAGTTAAATCAACAAGAGCT	GTCCGATGGCTACTATTAT
13	14702–15767	ACTAGCCACCAACCAAACAG	CGAAGTTTCATCAGGCAGAG
14	15663–16493	ATGATCTTAACCACACAGACC	GTTGTCCGATGATGATGAATG
15	16347–1128	CCTMCTRCTCACTCTTAT	CTATGCACGATATGCAT

Note: M refers to A and C, R refers to A and G.

**Figure 4:**
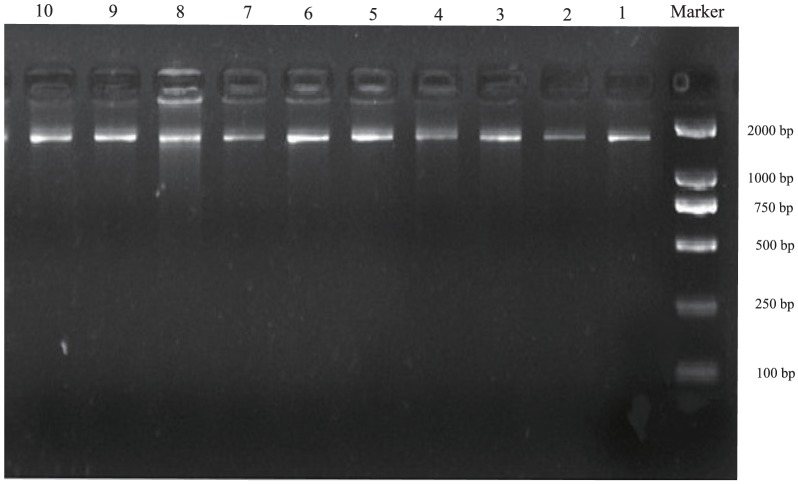
The typical amplification results (bands 1–10) of representative sequences from *Tadorna ferruginea* and *Aix galericulata*. Notes: bands 1–6 are the PCR amplification products used primers 6–11 of *Tadorna ferruginea*, and bands 7–10 are the PCR amplification products used primers 6–9 of *Aix galericulata*.

PCR amplifications were carried out in 50 µl volumes containing 100 ng template DNA, 5 µl of 10× reaction Buffer, 2 µl of 25 mM MgCl_2_, 4 µl of 2 mM dNTPs, 1 µl of each 10 mM primer, 0.5 U Taq DNA polymerase (Trans Taq-T DNA Polymerase, Beijing, China), and sterile doubly-distilled water to final volume. PCR amplification conditions follow: denaturation for 5 min at 94°C, followed by 30 cycles of denaturation for 30 s at 94°C, annealing for 30 s at 49–55°C (depending on primer combinations), elongation for 2 min at 72°C, and a final extension step of 10 min at 72°C. PCR products were examined using electrophoresis on a 1% agarose/TBE gel (Figure 4) and purified and bidirectionally sequenced by Sangon Biotech Co., Ltd. (Shanghai, China).

### Sequence analysis

Sequences were checked and assembled using the programs Seqman (DNASTAR, 2001), BioEdit, and Chromas 2.22, and then adjusted manually. PCGs were identified by comparison with the known complete mtDNA sequences of Anseriform birds using Sequin 11.0. The 22 tRNA genes were identified using the software package tRNA Scan-SE 1.21 (http://lowelab.ucsc.edu/tRNAscan-SE), and their cloverleaf secondary structures and anticodon sequences were determined using DNASIS (Ver.2.5, Hitachi Software Engineering). Two rRNAs were identified by comparison with complete mtDNA sequences of other Anseriformes available in GenBank. The complete mtDNA sequences have been deposited in GenBank under accession numbers KF437906 and KF684946.

### Phylogenetic analyses

Phylogenetic trees were estimated using ML and BI methods, to study the phylogeny of the Anseriformes. Corresponding *Gallus gallus* (NC_001323) sequence was used as outgroup. Phylogenetic trees were estimated for two cases: one based on the complete mtDNA of 16 Anseriforme species (Table 6), and another one based on a multiple sequence alignment of three mitogenome (Cyt b, ND2, and COI) sequences from 68 typical Anseriform species from GenBank (Table 7). Our previous research has shown that the combined gene sequence from Cyt b, ND2, and COI is suitable for resolving phylogenetic relationships among Anseriform species in the absence of sufficient complete mtDNA data [16].

**Table 6:** GenBank accession numbers for the 16 complete mtDNA of Anseriforme species in this study.

Species	Accession number	Species	Accession number
*Anas platyrhynchos*	NC_009684	*Aythya americana*	NC_000877
*Anas formosa*	NC_015482	*Branta canadensis*	NC_007011
*Anser anser*	NC_011196	*Cairina moschata*	NC_010965
*Anser albifrons*	NC_004539	*Cygnus columbianus*	NC_007691
*Anseranas semipalmata*	NC_005933	*Cygnus atratus*	NC_012843
*Dendrocygna javanica*	NC_012844	*Anas falcate*	KC759527
*Anser fabalis*	NC_016922	*Mergus squamatus*	NC_016723
*Aix galericulatata*	In this study	*Tadorna ferruginea*	In this study

**Table 7:** GenBank accession numbers (Cyt b, ND2 and COI) for the 68 Anseriforme species in this study.

Species	Accession number (Cyt b,ND2,COI)	Species	Accession number (Cyt b,ND2,COI)
*Anhima cornuta*	AY140735,AY140737,AY140729	*Anas falcata*	KC759527,KC759527,KC759527
*Anseranas semipalmata*	NC_005933,NC_005933,C_005933	*Anas poecilorhyncha*	EU914150,AF059143,JN703239
*Dendrocygna arcuata*	AF082061,U97735,U97739	*Anas discors*	EU914146,AF059128,DQ434285
*Dendrocygna eytoni*	EU585647,EU585710,U97733	*Anas acuta*	AF059055,AF059116,JN703180
*Dendrocygna viduata*	EU585649,EU585712,FJ027502	*Anas platalea*	AF059084,AF059144,FJ027099
*Dendrocygna javanica*	NC_012844,NC_012844,C_012844	*Anas clypeata*	EU914154,AF059174,DQ434274
*Anser albifrons*	NC_004539,NC_004539,C_004539	*Anas gibberifrons*	AF059076,AF059136,JQ174015
*Anser anser*	NC_011196,NC_011196,C_011196	*Anas bahamensis*	EU914147,AF059120,JQ174013
*Anser brachyrhynchus*	EU585614,EU585677,GU179004	*Anas querquedula*	AF059086,EU585673,GU571723
*Anser indicus*	EU585619,EU585682,GU179002	*Anas penelope*	AF059107,AF059167,JN703206
*Anser fabalis*	NC_016922,NC_016922,C_016922	*Anas platyrhynchos*	NC_009684,C_009684,C_009684
*Anser rossii*	EU914156,EU585683,DQ434539	*Anas laysanensis*	AF059078,AF059138,JF498830
*Anser canagica*	EU585615, EU585678, DQ432849	*Anas superciliosa*	FJ498963, AF059152, JN801396
*Branta bernicla*	EU585628,EU585691,DQ434344	*Anas formosa*	NC_015482,NC_015482,NC_015482
*Branta canadensis*	NC_007011,NC_007011,NC_007011	*Netta rufina*	EU585657,EU585720,GU571988
*Branta leucopsis*	EU585630,EU585693,GU179003	*Aythya ferina*	NC_000877,NC_000877,NC_000877
*Branta sandvicensis*	EU585632,EU585695,JF498832	*Aythya affinis*	EU585621,EU585684,DQ434310
*Coscoroba coscoroba*	EU585639, EU585702,FJ027452	*Aythya americana*	NC_000877,NC_000877,NC_000877
*Cygnus atratus*	NC_012843,NC_012843,C_012843	*Aythya marila*	EU585625,EU585688,DQ434333
*Cygnus columbianus*	NC_007691,NC_007691,NC_007691	*Aythya nyroca*	EU585626, EU585689, GQ481388
*Cygnus cygnus*	EU585643,EU585706,GU571854	*Somateria spectabilis*	EU585662,EU585725,DQ434103
*Cygnus olor*	EU585645,EU585708,GU571856	*Somateria mollissima*	AF515264,AF515268,DQ434751
*Tachyeres pteneres*	AF059112,JQ408332,JN802006	*Melanitta perspicillata*	EU585652,EU585715,DQ434655
*Sarkidiornis melanotos*	EU585660, EU585723, FJ028237	*Melanitta nigra*	AF515263,AF515267,AY666338
*Chloephaga picta*	AF515262,AF515266,FJ027353	*Bucephala albeola*	EU585633, EU585696, DQ434491
*Chloephaga poliocephala*	EU585637,EU585700,FJ027355	*Bucephala clangula*	AF515261, AF515265, EU525336
*Tadorna ferruginea*	In this study	*Bucephala islandica*	EU585635, EU585698, DQ434502
*Tadorna tadorna*	AF059113,AF059173,GU571650	*Mergellus albellus*	EU585653, EU585716, GU571480
*Cairina moschata*	NC_010965,NC_010965,NC_010965	*Lophodytes cucullatus*	EU585650, EU585713,AY666346
*Aix galericulata*	In this study	*Mergus serrator*	EU585655,EU585718,DQ434677
*Aix sponsa*	AF059053, AF059114, DQ434254	*Mergus squamatus*	NC_016723,NC_016723,NC_016723
*Lophonetta specularoides*	AF059102, JQ408334, FJ027113	*Mergus merganser*	EU585654, EU585717, DQ434672
*Amazonetta brasiliensis*	AF059054,AF059115,FJ027065	*Nomonyx dominicus*	AF119165,AY747864,FJ027905
*Anas strepera*	EU574791,AF059169,DQ434298	*Oxyura jamaicensis*	EU585658,EU585721,AY666448

Before phylogenetic tree estimations all 16 complete mtDNAs and the three concatenated data sets of 68 Anseriform species were aligned using ClustalX 1.8, followed by manual adjustment. Specifics of the phylogenetic tree estimation based on the three concatenated data sets among the 68 Anseriforme species follows: the three mitogenome sequences were translated into their corresponding amino acid sequences and saved into.meg format, then turned it into.nex format, and then remove each third position of every codon using MEGA 4.0. We then concatenated the three mitogenome sequences to each other under the each species bird name, saved the file in.meg format, and then turned it into nex form. ML analyses was performed in PAUP* 4.0b10 using TBR branch swapping (10 random addition sequences) and a general time-reversible model with invariant sites and among-site variation (GTR+I+Γ) that was selected as the best fit model of evolution using Modeltest (version 3.06) based on the AIC criterion. Internal ML tree branch support was evaluated using a bootstrap test with 100 iterations. Bayesian phylogenetic inference was done using MrBayes 3.1.2, with the same best-fit substitution model as that selected for the ML analysis. MrBayes 3.1.2 simultaneously initiates two Markov Chain Monte Carlo (MCMC) runs to provide additional confirmation of convergence of posterior probability distributions. Analyses were run for one million generations until the average standard deviation of split frequencies was less than 0.01, which indicated that convergence was reached. Chains were sampled every 1,000 generations.

## Supporting Information

Appendix S1 **Phylogenetic Classification of the Anseriformes.**
(DOC)
