# Supplementary material for: The Complete Mitochondrial Genome of Aix galericulata and Tadorna ferruginea: Bearings on Their Phylogenetic Position in the Anseriformes
Source: PLoS One. 2014 Nov 6;9(11):e109701. doi: 10.1371/journal.pone.0109701 (PMC4222781; doi:10.1371/journal.pone.0109701)
Supplement: Appendix S1 — Phylogenetic Classification of the Anseriformes. (DOC) [file pone.0109701.s001.doc]

**Appendix**

We reconstructed molecular phylogenetic analysis among some typical groups by 68 Anseriform birds based on concatenated sequences (Cyt b, ND2 and COI). The results are as follow:

Anseriformes

Family I: Anhimidae

*Anhimidae*

Family II: Anseranatidae

*Anseranas*

Family III: Anatidae

Subfamily I: Anatinae

Tribe I: Mergini

*Mergus*, *Mergellus*, *Melanitta*, *Bucephala*, *Lophodytes*

Tribe II: Somaterini

*Somateria*

Tribe III: Anatini

*Anas*, *Sarkidiornis*, *Tachyeres*, *Lophonetta*, *Amazonetta*

Tribe IV: Aythyini

*Aythya*, *Netta*

Subfamily II: Tadorninae

*Chloephaga*, *Tadorna*, *Cairina*, *Aix*, *Chloephaga*

Subfamily III: Anserinae

Tribe I: Anserini

*Branta*, *Anser*

Tribe II: Cygnini

*Oxyura*, *Nomonyx*, *Coscoroba*, *Cygnus*

Subfamily IV: Oxyurinae

*Nomonyx*, *Coscoroba*

Subfamily V: Dendrocygninae

*Dendrocygna*
